# Supplementary material for: Pyruvate Dehydrogenase Kinase 1 inhibition mediated oxidative phosphorylation enhancement in cartilage promotes osteoarthritis progression
Source: BMC Musculoskelet Disord. 2023 Jul 20;24:597. doi: 10.1186/s12891-023-06585-6 (PMC10357736; doi:10.1186/s12891-023-06585-6)
Supplement: Supplementary file 1 — Additional file 1. [file 12891_2023_6585_MOESM1_ESM.zip › KEGG.Gsea.1653622432464/KEGG_APOPTOSIS.html]

Details for gene set KEGG\_APOPTOSIS[GSEA]

|  || Dataset | 55457\_collapsed\_to\_symbols.cls.cls #OA\_versus\_normal.cls.cls #OA\_versus\_normal\_repos |
| Phenotype | cls.cls#OA\_versus\_normal\_repos |
| Upregulated in class | normal |
| GeneSet | KEGG\_APOPTOSIS |
| Enrichment Score (ES) | -0.3737051 |
| Normalized Enrichment Score (NES) | -1.5873159 |
| Nominal p-value | 0.0060698027 |
| FDR q-value | 0.16009048 |
| FWER p-Value | 0.694 |
Table: GSEA Results Summary

  

Fig 1: Enrichment plot: KEGG\_APOPTOSIS      
 Profile of the Running ES Score & Positions of GeneSet Members on the Rank Ordered List

  

| SYMBOL | TITLE | RANK IN GENE LIST | RANK METRIC SCORE | RUNNING ES | CORE ENRICHMENT || 1 | PRKACB | protein kinase cAMP-activated catalytic subunit beta [Source:HGNC Symbol;Acc:HGNC:9381] | 771 | 0.576 | -0.0379 | No |
| 2 | FADD | Fas associated via death domain [Source:HGNC Symbol;Acc:HGNC:3573] | 924 | 0.534 | -0.0292 | No |
| 3 | AKT1 | AKT serine/threonine kinase 1 [Source:HGNC Symbol;Acc:HGNC:391] | 1192 | 0.478 | -0.0316 | No |
| 4 | TNFSF10 | TNF superfamily member 10 [Source:HGNC Symbol;Acc:HGNC:11925] | 1241 | 0.468 | -0.0173 | No |
| 5 | AIFM1 | apoptosis inducing factor mitochondria associated 1 [Source:HGNC Symbol;Acc:HGNC:8768] | 1838 | 0.380 | -0.0492 | No |
| 6 | TNF | tumor necrosis factor [Source:HGNC Symbol;Acc:HGNC:11892] | 2098 | 0.350 | -0.0559 | No |
| 7 | PIK3CB | "phosphatidylinositol-4,5-bisphosphate 3-kinase catalytic subunit beta [Source:HGNC Symbol;Acc:HGNC:8976]" | 2186 | 0.340 | -0.0496 | No |
| 8 | ENDOD1 | endonuclease domain containing 1 [Source:HGNC Symbol;Acc:HGNC:29129] | 2242 | 0.334 | -0.0410 | No |
| 9 | CASP6 | caspase 6 [Source:HGNC Symbol;Acc:HGNC:1507] | 2427 | 0.311 | -0.0434 | No |
| 10 | NTRK1 | neurotrophic receptor tyrosine kinase 1 [Source:HGNC Symbol;Acc:HGNC:8031] | 3015 | 0.248 | -0.0796 | No |
| 11 | CASP3 | caspase 3 [Source:HGNC Symbol;Acc:HGNC:1504] | 3202 | 0.230 | -0.0852 | No |
| 12 | BAD | BCL2 associated agonist of cell death [Source:HGNC Symbol;Acc:HGNC:936] | 3788 | 0.178 | -0.1240 | No |
| 13 | PPP3CA | protein phosphatase 3 catalytic subunit alpha [Source:HGNC Symbol;Acc:HGNC:9314] | 3803 | 0.176 | -0.1183 | No |
| 14 | FAS | Fas cell surface death receptor [Source:HGNC Symbol;Acc:HGNC:11920] | 3828 | 0.173 | -0.1135 | No |
| 15 | FASLG | Fas ligand [Source:HGNC Symbol;Acc:HGNC:11936] | 3931 | 0.162 | -0.1152 | No |
| 16 | RELA | "RELA proto-oncogene, NF-kB subunit [Source:HGNC Symbol;Acc:HGNC:9955]" | 3942 | 0.161 | -0.1098 | No |
| 17 | ENDOG | endonuclease G [Source:HGNC Symbol;Acc:HGNC:3346] | 4091 | 0.147 | -0.1157 | No |
| 18 | NFKB1 | nuclear factor kappa B subunit 1 [Source:HGNC Symbol;Acc:HGNC:7794] | 4626 | 0.102 | -0.1534 | No |
| 19 | BIRC2 | baculoviral IAP repeat containing 2 [Source:HGNC Symbol;Acc:HGNC:590] | 5054 | 0.069 | -0.1841 | No |
| 20 | PPP3CB | protein phosphatase 3 catalytic subunit beta [Source:HGNC Symbol;Acc:HGNC:9315] | 5311 | 0.046 | -0.2023 | No |
| 21 | TP53 | tumor protein p53 [Source:HGNC Symbol;Acc:HGNC:11998] | 5511 | 0.032 | -0.2166 | No |
| 22 | BCL2 | BCL2 apoptosis regulator [Source:HGNC Symbol;Acc:HGNC:990] | 5609 | 0.023 | -0.2232 | No |
| 23 | TRADD | TNFRSF1A associated via death domain [Source:HGNC Symbol;Acc:HGNC:12030] | 5713 | 0.015 | -0.2307 | No |
| 24 | BID | BH3 interacting domain death agonist [Source:HGNC Symbol;Acc:HGNC:1050] | 5715 | 0.014 | -0.2302 | No |
| 25 | PRKAR1B | protein kinase cAMP-dependent type I regulatory subunit beta [Source:HGNC Symbol;Acc:HGNC:9390] | 5806 | 0.008 | -0.2369 | No |
| 26 | IKBKB | inhibitor of nuclear factor kappa B kinase subunit beta [Source:HGNC Symbol;Acc:HGNC:5960] | 5914 | 0.001 | -0.2452 | No |
| 27 | BIRC3 | baculoviral IAP repeat containing 3 [Source:HGNC Symbol;Acc:HGNC:591] | 5942 | -0.001 | -0.2473 | No |
| 28 | ATM | ATM serine/threonine kinase [Source:HGNC Symbol;Acc:HGNC:795] | 6179 | -0.019 | -0.2650 | No |
| 29 | MYD88 | MYD88 innate immune signal transduction adaptor [Source:HGNC Symbol;Acc:HGNC:7562] | 6638 | -0.052 | -0.2987 | No |
| 30 | DFFA | DNA fragmentation factor subunit alpha [Source:HGNC Symbol;Acc:HGNC:2772] | 6654 | -0.053 | -0.2978 | No |
| 31 | BCL2L1 | BCL2 like 1 [Source:HGNC Symbol;Acc:HGNC:992] | 6698 | -0.055 | -0.2991 | No |
| 32 | PPP3CC | protein phosphatase 3 catalytic subunit gamma [Source:HGNC Symbol;Acc:HGNC:9316] | 7031 | -0.082 | -0.3218 | No |
| 33 | PRKAR2B | protein kinase cAMP-dependent type II regulatory subunit beta [Source:HGNC Symbol;Acc:HGNC:9392] | 7034 | -0.082 | -0.3188 | No |
| 34 | PIK3CD | "phosphatidylinositol-4,5-bisphosphate 3-kinase catalytic subunit delta [Source:HGNC Symbol;Acc:HGNC:8977]" | 7405 | -0.111 | -0.3434 | No |
| 35 | NGF | nerve growth factor [Source:HGNC Symbol;Acc:HGNC:7808] | 7572 | -0.125 | -0.3515 | No |
| 36 | PIK3CA | "phosphatidylinositol-4,5-bisphosphate 3-kinase catalytic subunit alpha [Source:HGNC Symbol;Acc:HGNC:8975]" | 7733 | -0.136 | -0.3587 | No |
| 37 | CSF2RB | colony stimulating factor 2 receptor subunit beta [Source:HGNC Symbol;Acc:HGNC:2436] | 7826 | -0.143 | -0.3604 | No |
| 38 | IL1A | interleukin 1 alpha [Source:HGNC Symbol;Acc:HGNC:5991] | 7842 | -0.144 | -0.3560 | No |
| 39 | PIK3CG | "phosphatidylinositol-4,5-bisphosphate 3-kinase catalytic subunit gamma [Source:HGNC Symbol;Acc:HGNC:8978]" | 7953 | -0.152 | -0.3588 | No |
| 40 | AKT3 | AKT serine/threonine kinase 3 [Source:HGNC Symbol;Acc:HGNC:393] | 8008 | -0.156 | -0.3570 | No |
| 41 | MAP3K14 | mitogen-activated protein kinase kinase kinase 14 [Source:HGNC Symbol;Acc:HGNC:6853] | 8100 | -0.163 | -0.3578 | No |
| 42 | CAPN2 | calpain 2 [Source:HGNC Symbol;Acc:HGNC:1479] | 8114 | -0.164 | -0.3525 | No |
| 43 | CASP9 | caspase 9 [Source:HGNC Symbol;Acc:HGNC:1511] | 8142 | -0.166 | -0.3482 | No |
| 44 | TNFRSF1A | TNF receptor superfamily member 1A [Source:HGNC Symbol;Acc:HGNC:11916] | 8196 | -0.171 | -0.3458 | No |
| 45 | TRAF2 | TNF receptor associated factor 2 [Source:HGNC Symbol;Acc:HGNC:12032] | 8322 | -0.180 | -0.3486 | No |
| 46 | CYCS | "cytochrome c, somatic [Source:HGNC Symbol;Acc:HGNC:19986]" | 8410 | -0.186 | -0.3482 | No |
| 47 | CAPN1 | calpain 1 [Source:HGNC Symbol;Acc:HGNC:1476] | 8500 | -0.194 | -0.3477 | No |
| 48 | CHP2 | calcineurin like EF-hand protein 2 [Source:HGNC Symbol;Acc:HGNC:24927] | 8573 | -0.199 | -0.3456 | No |
| 49 | XIAP | X-linked inhibitor of apoptosis [Source:HGNC Symbol;Acc:HGNC:592] | 8750 | -0.212 | -0.3511 | No |
| 50 | PIK3R3 | phosphoinositide-3-kinase regulatory subunit 3 [Source:HGNC Symbol;Acc:HGNC:8981] | 8801 | -0.217 | -0.3467 | No |
| 51 | PIK3R5 | phosphoinositide-3-kinase regulatory subunit 5 [Source:HGNC Symbol;Acc:HGNC:30035] | 8804 | -0.217 | -0.3385 | No |
| 52 | CASP7 | caspase 7 [Source:HGNC Symbol;Acc:HGNC:1508] | 9232 | -0.251 | -0.3621 | Yes |
| 53 | BAX | "BCL2 associated X, apoptosis regulator [Source:HGNC Symbol;Acc:HGNC:959]" | 9323 | -0.257 | -0.3592 | Yes |
| 54 | IRAK1 | interleukin 1 receptor associated kinase 1 [Source:HGNC Symbol;Acc:HGNC:6112] | 9377 | -0.262 | -0.3533 | Yes |
| 55 | IL1RAP | interleukin 1 receptor accessory protein [Source:HGNC Symbol;Acc:HGNC:5995] | 9395 | -0.263 | -0.3444 | Yes |
| 56 | CASP10 | caspase 10 [Source:HGNC Symbol;Acc:HGNC:1500] | 9404 | -0.264 | -0.3349 | Yes |
| 57 | IL3 | interleukin 3 [Source:HGNC Symbol;Acc:HGNC:6011] | 9850 | -0.306 | -0.3578 | Yes |
| 58 | TNFRSF10D | TNF receptor superfamily member 10d [Source:HGNC Symbol;Acc:HGNC:11907] | 10055 | -0.326 | -0.3611 | Yes |
| 59 | PRKAR1A | protein kinase cAMP-dependent type I regulatory subunit alpha [Source:HGNC Symbol;Acc:HGNC:9388] | 10093 | -0.330 | -0.3513 | Yes |
| 60 | EXOG | exo/endonuclease G [Source:HGNC Symbol;Acc:HGNC:3347] | 10266 | -0.347 | -0.3513 | Yes |
| 61 | CASP8 | caspase 8 [Source:HGNC Symbol;Acc:HGNC:1509] | 10442 | -0.366 | -0.3508 | Yes |
| 62 | PRKACG | protein kinase cAMP-activated catalytic subunit gamma [Source:HGNC Symbol;Acc:HGNC:9382] | 10466 | -0.370 | -0.3384 | Yes |
| 63 | PRKACA | protein kinase cAMP-activated catalytic subunit alpha [Source:HGNC Symbol;Acc:HGNC:9380] | 10611 | -0.384 | -0.3348 | Yes |
| 64 | PRKAR2A | protein kinase cAMP-dependent type II regulatory subunit alpha [Source:HGNC Symbol;Acc:HGNC:9391] | 10664 | -0.389 | -0.3239 | Yes |
| 65 | TNFRSF10C | TNF receptor superfamily member 10c [Source:HGNC Symbol;Acc:HGNC:11906] | 10731 | -0.397 | -0.3137 | Yes |
| 66 | IRAK4 | interleukin 1 receptor associated kinase 4 [Source:HGNC Symbol;Acc:HGNC:17967] | 10874 | -0.413 | -0.3089 | Yes |
| 67 | CHP1 | calcineurin like EF-hand protein 1 [Source:HGNC Symbol;Acc:HGNC:17433] | 10940 | -0.423 | -0.2976 | Yes |
| 68 | PIK3R2 | phosphoinositide-3-kinase regulatory subunit 2 [Source:HGNC Symbol;Acc:HGNC:8980] | 10956 | -0.425 | -0.2824 | Yes |
| 69 | IKBKG | inhibitor of nuclear factor kappa B kinase regulatory subunit gamma [Source:HGNC Symbol;Acc:HGNC:5961] | 10986 | -0.428 | -0.2682 | Yes |
| 70 | TNFRSF10B | TNF receptor superfamily member 10b [Source:HGNC Symbol;Acc:HGNC:11905] | 11020 | -0.433 | -0.2540 | Yes |
| 71 | PIK3R1 | phosphoinositide-3-kinase regulatory subunit 1 [Source:HGNC Symbol;Acc:HGNC:8979] | 11099 | -0.443 | -0.2430 | Yes |
| 72 | CHUK | component of inhibitor of nuclear factor kappa B kinase complex [Source:HGNC Symbol;Acc:HGNC:1974] | 11624 | -0.521 | -0.2638 | Yes |
| 73 | PRKX | protein kinase X-linked [Source:HGNC Symbol;Acc:HGNC:9441] | 11634 | -0.522 | -0.2444 | Yes |
| 74 | RIPK1 | receptor interacting serine/threonine kinase 1 [Source:HGNC Symbol;Acc:HGNC:10019] | 11953 | -0.584 | -0.2467 | Yes |
| 75 | CFLAR | CASP8 and FADD like apoptosis regulator [Source:HGNC Symbol;Acc:HGNC:1876] | 12079 | -0.614 | -0.2328 | Yes |
| 76 | IL1B | interleukin 1 beta [Source:HGNC Symbol;Acc:HGNC:5992] | 12105 | -0.622 | -0.2108 | Yes |
| 77 | IL3RA | interleukin 3 receptor subunit alpha [Source:HGNC Symbol;Acc:HGNC:6012] | 12380 | -0.703 | -0.2051 | Yes |
| 78 | IRAK3 | interleukin 1 receptor associated kinase 3 [Source:HGNC Symbol;Acc:HGNC:17020] | 12383 | -0.703 | -0.1781 | Yes |
| 79 | IL1R1 | interleukin 1 receptor type 1 [Source:HGNC Symbol;Acc:HGNC:5993] | 12419 | -0.713 | -0.1533 | Yes |
| 80 | DFFB | DNA fragmentation factor subunit beta [Source:HGNC Symbol;Acc:HGNC:2773] | 12420 | -0.714 | -0.1258 | Yes |
| 81 | PPP3R1 | "protein phosphatase 3 regulatory subunit B, alpha [Source:HGNC Symbol;Acc:HGNC:9317]" | 12653 | -0.829 | -0.1120 | Yes |
| 82 | APAF1 | apoptotic peptidase activating factor 1 [Source:HGNC Symbol;Acc:HGNC:576] | 12812 | -0.993 | -0.0860 | Yes |
| 83 | NFKBIA | NFKB inhibitor alpha [Source:HGNC Symbol;Acc:HGNC:7797] | 12817 | -1.001 | -0.0477 | Yes |
| 84 | AKT2 | AKT serine/threonine kinase 2 [Source:HGNC Symbol;Acc:HGNC:392] | 12896 | -1.404 | 0.0003 | Yes |
Table: GSEA details [plain text format]

  

Fig 2: KEGG\_APOPTOSIS      
 Blue-Pink O' Gram in the Space of the Analyzed GeneSet

  

Fig 3: KEGG\_APOPTOSIS: Random ES distribution      
 Gene set null distribution of ES for **KEGG\_APOPTOSIS**
